# When Is It Safe to Fly? Early Air Travel After Small Traumatic Pneumothorax

**DOI:** 10.1002/rcr2.70516

**Published:** 2026-02-16

**Authors:** Arabella T. Patrick, Alasdair B. Patrick

**Affiliations:** ^1^ School Of Medical Sciences, Faculty of Medicine and Health University of Sydney Sydney New South Wales Australia; ^2^ MacMurray Institute Auckland New Zealand

**Keywords:** air travel, clinical guidelines, pneumothorax, traumatic pneumothorax, treatment delay

## Abstract

Commercial air travel exposes passengers to reduced cabin pressures, causing intrathoracic gas volume to expand (Boyle's law). Guidelines recommend waiting 7–14 days after radiographic resolution of a pneumothorax before flying; however, such recommendations may not reflect emerging evidence for select cases. We report a case of a healthy 51‐year‐old man with a small (< 10%) traumatic pneumothorax who flew domestically and internationally within 4 and 9 days of diagnosis against medical advice. He remained asymptomatic, with serial chest X‐rays indicating stability and eventual resolution of the pneumothorax. The patient demonstrated clinical and radiographic stability despite commercial cabin pressure changes and high‐altitude activity. The presented case supports and extends a growing body of research, suggesting that patients with normal oxygen saturation on room air may safely tolerate air travel with a small, stable, traumatic pneumothorax. This case highlights the potential need for individualised risk assessment when advising travel delays.

## Graphical abstract

Existing guidelines recommend delayed commercial air travel after pneumothorax, due to the risk of gas expansion and potentially fatal deterioration. Patients are advised to delay travel for 7–14 days after radiographic confirmation of a full resolution. Such recommendations are based on limited evidence and observational data. The presented case supports and extends a growing body of research, suggesting that patients with normal oxygen saturation on room air may safely tolerate air travel with a small, stable, traumatic pneumothorax. This case highlights the potential need for individualised risk assessment when advising travel delays.

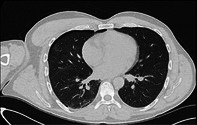

## 1 Introduction

Commercial aircraft cabins are pressurised with compressed ambient air from the jet engines. Cabin pressure typically corresponds to an altitude of 8000 ft. (2440 m), where the ambient pressure is roughly 75% of that at sea level [1]. According to Boyle's law, the volume (V) of a gas is inversely proportional to the ambient pressure (P) (P_1_V_1_ = P_2_V_2_). Theoretically, therefore, when an aeroplane ascends, barometric pressure decreases, and trapped gas within closed body spaces (i.e., pleural cavity containing air from a pneumothorax) expands proportionally [2]. The volume expansion in the chest cavity will impair ventilation relative to sea level, potentially impacting the patient's respiratory function [2].

Existing guidelines recommend delayed commercial air travel after pneumothorax, due to the risk of gas expansion and potentially fatal deterioration [1]. Patients are advised to delay travel for 7–14 days after radiographic confirmation of a full resolution [2]. Such recommendations are based on limited evidence and observational data. However, emerging evidence suggests that selected patients with small, stable traumatic pneumothorax and preserved oxygenation may safely tolerate earlier air travel [3, 4, 5]. The presented case supports and extends this growing body of evidence, highlighting the potential need for individualised risk assessment, rather than blanket delays.

## 2 Case Report

**FIGURE 1:**
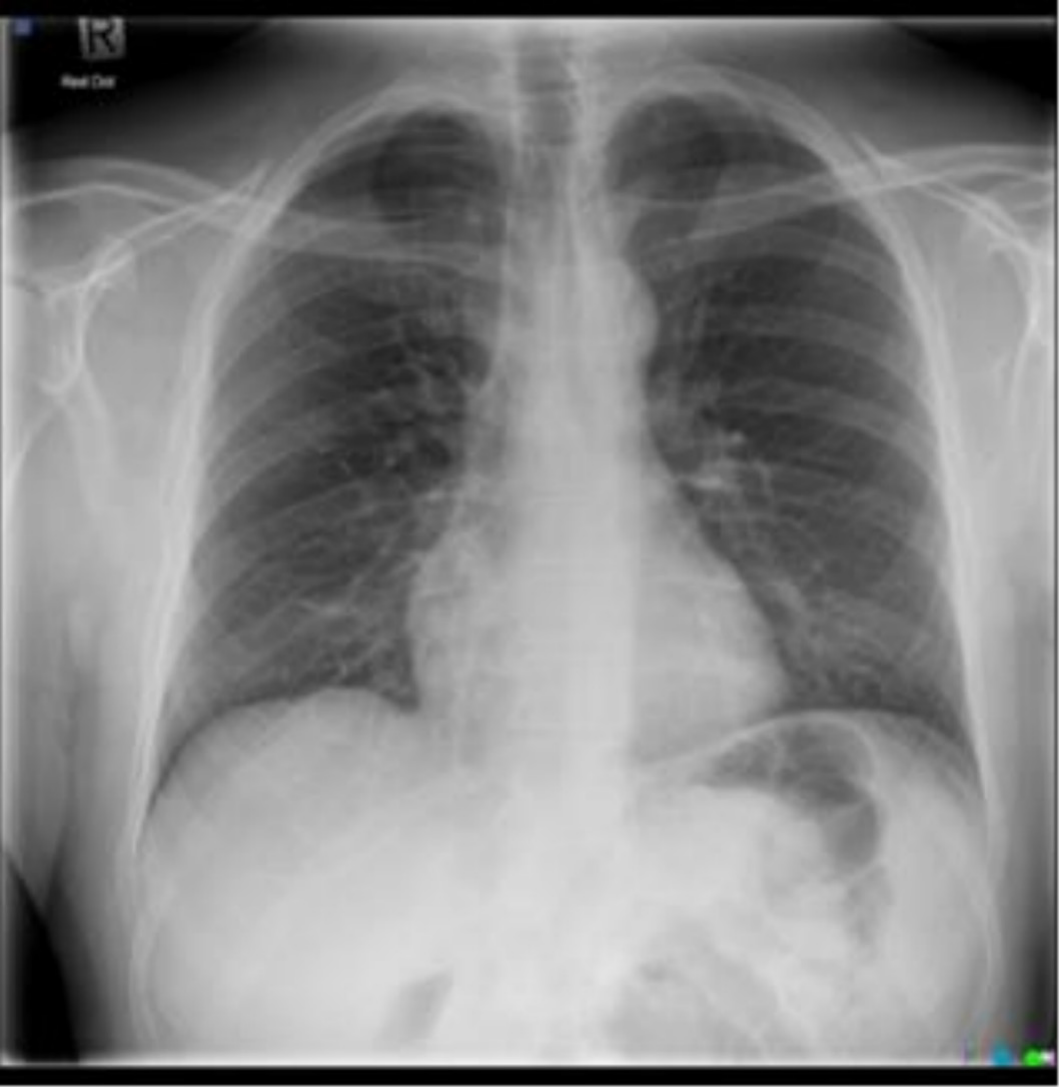
Chest X‐Ray, day of injury, small right apical pneumothorax with no obvious bony injury.

**FIGURE 2:**
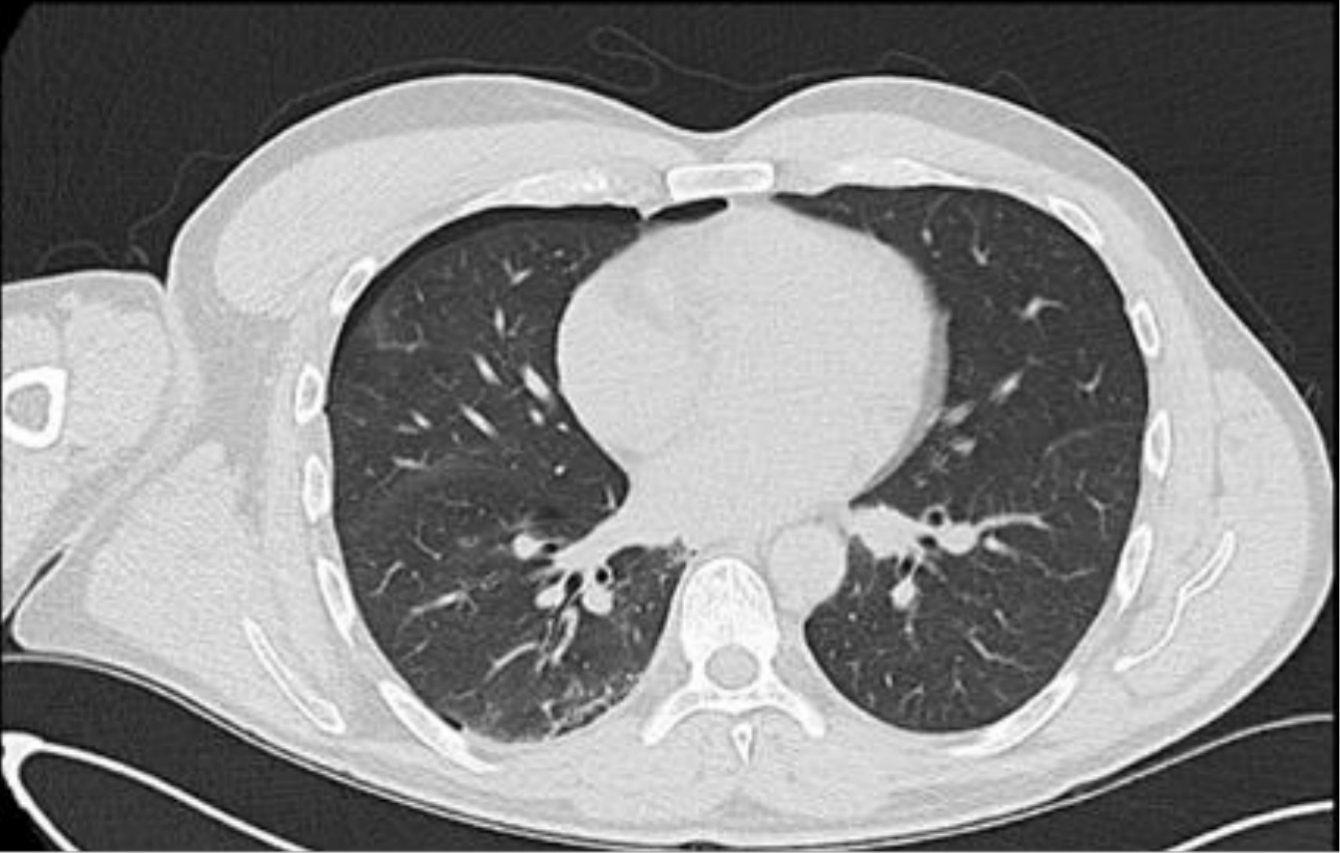
CT Chest, Day of injury, small < 10% pneumothorax on the right side.

**FIGURE 3:**
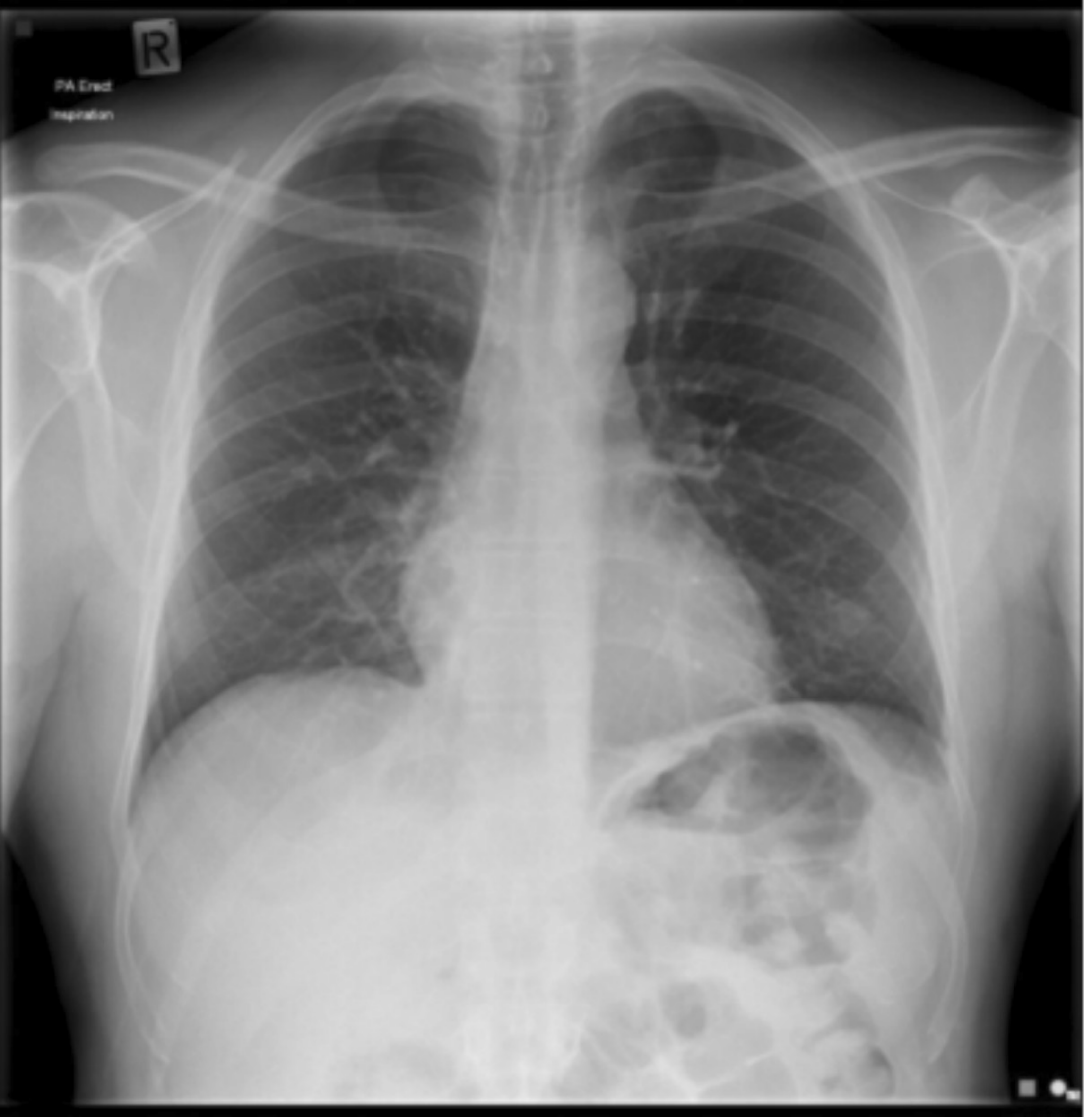
Chest X‐Ray, 1‐day post‐injury, stable right‐sided pneumothorax.

A 51‐year‐old male, otherwise healthy and physically active, presented to the emergency department (ED) with a small pneumothorax (< 10%) following a mountain biking accident in New Zealand (NZ). The patient was a non‐smoker with no prior lung disease or significant family history. No fractures or additional bony injuries were identified on the chest X‐Ray (CXR) or CT scan (Figures 1 and 2, respectively). Clinically, other than an initial brief period of mild shortness of breath, the patient was ambulatory, asymptomatic, and well, with normal chest signs. Oxygen saturations on room air were 100%. Arterial blood gas analysis was not performed. An intercostal drain was suggested by the emergency physician; however, the patient elected conservative management due to concerns about introducing a foreign body into the chest wall. On Day 2, a repeat CXR confirmed the size of the pneumothorax as stable (Figure 3). The patient was advised by the ED to perform a repeat CXR after a week and not to fly until medically cleared as per accepted guidelines.

**FIGURE 4:**
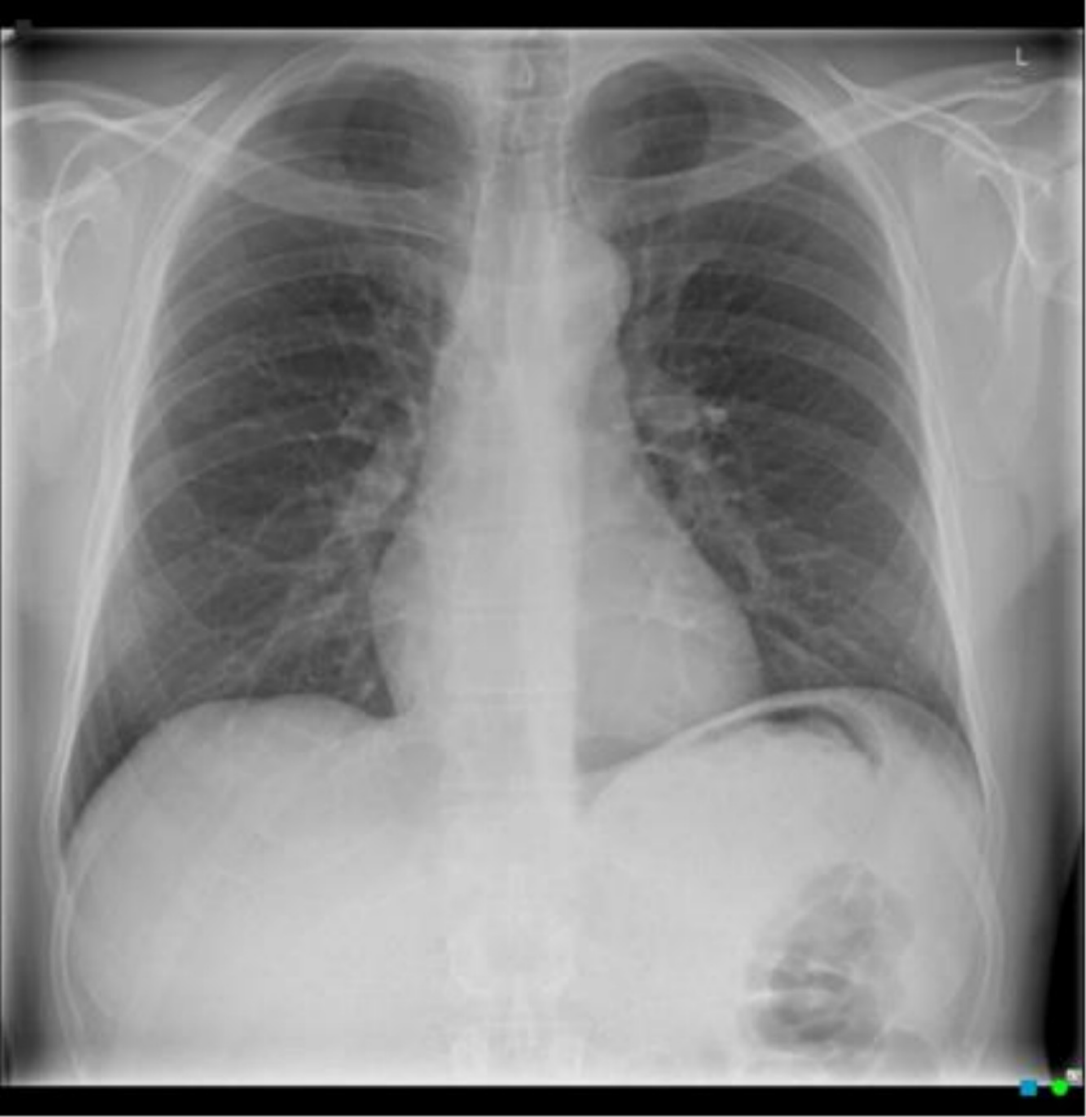
Chest X‐Ray, 24‐days post‐injury, resolution of previous pneumothorax.

Despite medical advice, on Day 6, the patient took a two‐hour commercial flight within NZ. During the flight, the patient was asymptomatic, suggesting the pneumothorax was not exacerbated. On Day 9, the patient took two subsequent commercial flights from NZ to Japan, the flights lasting 11 and 2 h respectively, with no symptom development. During the next 10 days, the patient engaged in frequent high‐altitude activity whilst skiing, reaching a maximum elevation of 1606 m on 132 occasions. He remained asymptomatic throughout this period. On Day 25, the patient returned to NZ via the same two commercial flights, remaining asymptomatic throughout. Upon arrival, a repeat CXR confirmed a complete resolution of the pneumothorax (Figure 4).

## 3 Discussion

According to joint reports, it is estimated that global passenger traffic will reach 9.5 billion in 2025, marking the highest number in aviation history [6]. Primary pneumothoraces are a relatively common condition, with an estimated annual incidence of 7.4–18 people per 100,000 in men and 1.2 to 6 people per 100,000 in women [7]. The Air Medical Journal review revealed that most medical societies recommend a waiting period of two weeks after full radiographic resolution of a pneumothorax before commercial air travel [2]. However, the current guidelines are conservative and may not fully reflect evolving evidence.

Recent studies suggest that earlier flying may be safe in selected cases [3, 4, 5]. Sacco and Calero [3] reported a retrospective case series demonstrating no adverse outcomes in patients flying within 9 days of chest tube removal, including those with small residual pneumothoraces. Similarly, Majercik et al. [4] demonstrated tolerance of hypobaric conditions in patients with adequate oxygen saturation, supporting the clinical relevance of preserved pulmonary reserve in travel recommendations. In addition, Tam et al. [5] evaluated air travel safety after percutaneous transthoracic needle biopsy. No patients reported any in‐flight medical events that required emergent medical attention or flight diversion. The study concluded that air travel can occur safely before radiographic resolution of pneumothorax as soon as 24 h after biopsy, adding to existing research that may support earlier travel.

Our findings align with Sacco et al. [3], Majercik et al. [4] and Tam et al. [5], with the patient breaking recommendations yet remaining asymptomatic through multiple commercial air flights and repeated high‐altitude exposure. This case fits the growing pattern of evidence raising critical questions surrounding the guidelines for commercial air travel after pneumothorax [3, 4, 5]. The patient's asymptomatic state during and after the commercial flights taken on Days 4 and 9 (well before the 7‐day delay period) suggests that changes in cabin pressure did not induce re‐expansion or tension phenomena in the pneumothorax.

Based on this case and existing literature, an individualised risk assessment for air travel following traumatic pneumothorax may consider the following factors:

- **Aetiology of Pneumothorax** ‐ The underlying cause of pneumothorax is an important determinant of risk. A small traumatic pneumothorax, in the absence of underlying lung disease or associated rib fracture, may carry lower risk of exacerbation during air travel compared with a spontaneous pneumothorax or one occurring in the context of chronic lung disease. In the present case, the lack of bony injury and underlying lung condition may have reduced the likelihood of further complications and lung damage.
- **Pneumothorax Size and Radiographic Stability** ‐ Both the size of the pneumothorax and its stability on serial imaging should be considered. Several studies suggest that early air travel may be tolerated in select patients with small, stable traumatic pneumothoraces, including those described by Sacco and Calero and Majercik et al. [3, 4]. Small pneumothoraces that remain stable or show interval improvement on chest imaging over 24–48 h may be less likely to undergo clinically significant expansion at cabin altitude. This is explained by Boyle's law, which predicts the degree of expansion of air in an enclosed pleural space. Assuming constant temperature, with a typical commercial aeroplane cabin altitude of 8000 ft., the expansion factor compared to sea level would be approximately 35% or an increased factor of 1.35. Therefore, in theory, a pneumothorax occupying 10% of the pleural space would increase in size to 13.5%. This equates to a 3.5% decrease in the volume of one lung or roughly a 1.75% reduction in total lung capacity; changes that are likely to be clinically insignificant in otherwise healthy individuals. Determining a particular threshold below which a pneumothorax may be considered physiologically negligible for air travel safety remains an important area for future research. Establishing such parameters would help to define clear, evidence‐based guidelines.
- **Baseline Oxygenation and Clinical Status** ‐ Pre‐flight risk assessment should consider the baseline clinical status of the patient, including oxygen saturation on room air, symptom burden, and overall pulmonary reserve. Preserved oxygenation saturation (> 93%–94%) without supplemental oxygen and an absence of exertional or resting dyspnoea may indicate appropriate reserve to tolerate mild hypobaric hypoxia during commercial air travel. Emerging evidence from hypobaric chamber studies, along with the presented case, may support this recommendation [4].
- **Planned Flight Characteristics** ‐ The planned flight characteristics, including flight duration, the availability of in‐flight medical support, and the feasibility of medical diversion, should be considered. During short‐haul flights, cabin pressures change quickly, and therefore, passengers can experience brief periods of hypoxia. Once the aircraft has reached 8000 ft., cabin pressure stabilises. Long‐haul flights have more gradual pressure changes; however, low‐grade hypoxia may be prolonged due to the time in the air. Further, during long‐haul flights, prolonged supplementary oxygen is not available, and access to advanced medical care is limited, which may increase the consequences of in‐flight deterioration even if the absolute risk is low.
  The aforenoted factors should not replace existing guidelines but may assist clinicians in identifying patients who may benefit from an individualised, risk‐stratified approach to air travel recommendations following pneumothorax.

Traditionally, aviation medicine has adopted a conservative approach to medical clearance, appropriately reflecting the constraints of the in‐flight environment. Limited medical resources, restricted access to advanced interventions, lack of supplemental oxygen, and the logistical and financial consequences of flight diversion mean clinical deterioration at altitude is difficult to manage. Events of deterioration may affect not only the individual patient but also other passengers and airline operations. Due to this, existing guidelines are designed to minimise the likelihood of in‐flight medical emergencies, aiming to protect both the patient's safety and the aviation system. Whilst a conservative approach is appropriate in cases of uncertainty, an individualised risk assessment informed by aetiology, size, clinical and radiographic stability, baseline oxygen levels and planned flight characteristics, when applied cautiously, may help to reduce unnecessary travel restrictions in selected low‐risk patients.

To conclude, this case adds to emerging evidence suggesting that selected patients may safely undertake commercial air travel shortly after the development of a small, stable pneumothorax, despite traditional recommendations for a 7‐day delay following radiographic resolution. The patient remained asymptomatic during both short and long‐haul flights and on additional high‐altitude exposure, with no radiographic or clinical exacerbation observed. Current aviation guidelines are necessarily conservative; however, they may not fully reflect the growing body of clinical evidence. Individualised risk assessment may be appropriate in place of uniform travel delays in asymptomatic patients with small post‐traumatic pneumothoraces providing patients are normoxic on room air and demonstrate radiographic stability over time. As a single case, these findings are not generalisable and do not supersede current guidelines; however, they contribute to a growing literature that may support guideline adjustment. Further prospective studies are warranted to define patient‐specific risk factors and refine suggestions for air travel, with the goal to reduce patient burden without compromising safety. These could be performed outside of aviation, potentially making use of the number of inhabited places above 8000 ft., or by further study using medical barometric pressure centres.

## Disclosure

Artificial intelligence tools were used to assist with language editing and structural refinement of the manuscript. All clinical content, analysis and conclusions were authored and verified by the authors.

## Consent

The authors declare that written informed consent was obtained for the publication of this manuscript and accompanying images using the consent form provided by the Journal.

## Conflicts of Interest

The authors declare no conflicts of interest.

## Data Availability

Data sharing not applicable to this article as no datasets were generated or analysed during the current study.
